# Alcohol consumption: context and association with mortality in Switzerland

**DOI:** 10.1007/s00394-022-03073-w

**Published:** 2022-12-24

**Authors:** Flurina Suter, Giulia Pestoni, Janice Sych, Sabine Rohrmann, Julia Braun

**Affiliations:** 1 Division of Chronic Disease Epidemiology, Epidemiology, Biostatistics and Prevention Institute (EBPI), University of Zurich, Zurich, Switzerland; 2 Nutrition Group, Health Department, Swiss Distance University of Applied Sciences, Zurich, Switzerland; 3 Institute of Food and Beverage Innovation, ZHAW School of Life Sciences and Facility Management, Waedenswil, Switzerland; 4 Divisions of Epidemiology and Biostatistics, Epidemiology, Biostatistics and Prevention Institute, University of Zurich, Zurich, Switzerland

**Keywords:** Alcohol consumption, Non-communicable disease mortality, menuCH study, Spatial analysis

## Abstract

**Purpose:**

Non-communicable diseases generate the largest number of avoidable deaths often caused by risk factors such as alcohol, smoking, and unhealthy diets. Our study investigates the association between amount and context of alcohol consumption and mortality from major non-communicable diseases in Switzerland.

**Methods:**

Generalized linear regression models were fitted on data of the cross-sectional population-based National Nutrition Survey menuCH (2014–2015, *n* = 2057). Mortality rates based on the Swiss mortality data (2015–2018) were modeled by the alcohol consumption group considering the amount and context (i.e., during or outside mealtime) of alcohol consumption and potential confounders. The models were checked for spatial autocorrelation using Moran’s *I* statistic. Integrated nested Laplace approximation (INLA) models were fitted when evidence for missing spatial information was found.

**Results:**

Higher mortality rates were detected among drinkers compared to non-drinkers for all-cancer (rate ratio (RR) ranging from 1.01 to 1.07) and upper aero-digestive tract cancer (RR ranging from 1.15 to 1.20) mortality. Global Moran’s *I* statistic revealed spatial autocorrelation at the Swiss district level for all-cancer mortality. An INLA model led to the identification of three districts with a significant decrease and four districts with a significant increase in all-cancer mortality.

**Conclusion:**

Significant associations of alcohol consumption with all-cancer and upper aero-digestive tract cancer mortality were detected. Our study results indicate the need for further studies to improve the next alcohol-prevention scheme and to lower the number of avoidable deaths in Switzerland.

**Supplementary Information:**

The online version contains supplementary material available at 10.1007/s00394-022-03073-w.

## Introduction

Non-communicable diseases (NCDs), such as cardiovascular diseases (CVD), diabetes, and cancers are the leading causes of death [1]. Moreover, NCDs cause most avoidable deaths often related to well-known risk factors, such as alcohol consumption, tobacco, and unhealthy diets [2, 3].

Previous studies have investigated the association of alcohol consumption with non-communicable diseases. However, the relationship between amount of alcohol consumption and health risks are not fully resolved yet [4–11]. A Swiss study found a J-shaped curve association between coronary heart disease risk and alcohol consumption, indicating a protective effect of moderate alcohol intake [12]. Nevertheless, other studies have shown the opposite effect, reporting an association of moderate alcohol consumption with liver disease and specific cancer site risk [4, 13, 14]. Alcohol is known to be carcinogenic for humans and has been classified by the International Agency for Research on Cancer (IARC) as a group 1 carcinogen [15]. It is a drug with a toxic effect on the human’s organs and tissue and its consumption can lead to psychoactive effects, which can in turn lead to injuries and accidents [16]. The IARC classifies alcoholic beverages as a carcinogen with sufficient evidence for the following cancer sites: oral cavity, pharynx, larynx, upper digestive tract, esophagus, colorectal, liver, bile duct and breast (in women) and with suggestive evidence for stomach, lung, and pancreatic cancer [17, 18].

The safe-drinking guideline by the Swiss Federal Commission for Alcohol Issues (EKAL) recommends to not exceed a maximum daily intake of 12 g for a healthy woman and 24 g for a healthy man [19]. The recommendation for women corresponds to a maximum of one standard glass which is about 3 dl beer, 1 dl wine, or 0.25 dl liquor and twice this amount for men [19]. In 2017, 8.4% of all deaths in Switzerland between 15 and 74 years of age were caused by alcohol consumption, indicating an urgent need for a targeted alcohol-prevention scheme [20].

Previous studies suppose that not only the amount but also the context of alcohol consumption influences the risk of non-communicable disease [21, 22]. Evidence suggests that the consumption of alcoholic beverages without a meal might be more detrimental to health than consumption of alcohol with a meal [21, 22].

The aim of our study was to investigate amount and context of alcohol consumption, using dietary, sociodemographic, anthropometric, and lifestyle data from the first National Nutrition Survey, the menuCH study. Since the relationship between alcohol consumption and health risks are not fully resolved yet, we investigated the association between amount and context of alcohol consumption and mortality from major non-communicable diseases in Switzerland.

## Methods

The structure of this report was based on the STROBE-nut guidelines [23]. The data used for this study were combined from three different sources: the menuCH study (2014–2015), the Swiss population census data (2015–2018), and the Swiss mortality data (2015–2018). The three data sources were combined at the district level.

### Study design and participants of menuCH

The menuCH study is a cross-sectional population-based study conducted between January 2014 and February 2015 in ten centers across Switzerland [24, 25]. It included two 24-h dietary recalls (24HDR) and one questionnaire about sociodemographic, dietary, and lifestyle factors [25, 26]. The first 24HDR was conducted on-site in one of the centers, and the second one took place two to six weeks later by telephone [25].

In collaboration with the Federal Statistical Office (FSO), a target sample of 4,627,878 Swiss residents was drawn. The stratified, random sample included adults of 18–75 years of age, which represented both sexes, five age categories (18–29, 30–39, 40–49, 50–64, and ≥ 65 years old), the three main language regions in Switzerland (CH-German, CH-French, and CH-Italian), and the twelve most populated Swiss cantons of the seven major regions [25, 27].

From the source population consisting of 13,606 participants, 5496 participants were successfully contacted and eligible for the study [28]. Excluded from the study were 3410 non-responders and 29 participants who did not complete the dietary assessment [28]. The final study sample consisted of 2057 participants [28]. Detailed information on the study recruitment are presented in Online Resource Fig. S1.

### Anthropometric, lifestyle and demographic factors

The participants’ lifestyle and demographic factors were derived from the self-administered questionnaire [26]. The following variables were used in our study: sex (male, female), age (divided into eleven categories: 18–24, 25–29, 30–34, 35–39, 40–44, 45–49, 50–54, 55–59, 60–64, 65–69, 70–75 years old), Swiss language region (CH-German, CH-French, CH-Italian), education level (primary, secondary, tertiary), physical activity (low, moderate, high; based on the short-form International Physical Activity Questionnaire (IPAQ) definitions [29]), and smoking habits (never, former, current). The anthropometric factors of height, weight, and hip circumference were measured during the first 24HDR by trained interviewers following the WHO-MONICA protocol [25, 30]. The participants’ body weight and height were used to calculate their body mass index (BMI). For pregnant or lactating women, their self-reported weight before pregnancy was used to calculate the BMI. According to the World Health Organization (WHO) definitions, the participants were classified as ‘underweight’ (BMI < 18.5 kg/m^2^), ‘normal weight’ (18.5 kg/m^2^ ≤ BMI < 25.0 kg/m^2^), ‘overweight’ (25.0 kg/m^2^ ≤ BMI < 30.0 kg/m^2^) or ‘obese’ (BMI ≥ 30 kg/m^2^) [31].

### Dietary assessment

The dietary data were assessed by two 24HDR, which were distributed over all weekdays and seasons [32]. The data were collected by trained dietitians who used the trilingual Swiss version (0.2014.02.27) of the automated software GloboDiet® (GD, formerly EPIC-Soft^®^, IARC, Lyon, France [33, 34]), which was adapted by the Federal Food Safety and Veterinary Office, Bern, Switzerland. To facilitate the quantification of consumed amounts, a book with 119 series of six graduated portion-size pictures [35] and about 60 actual household measures were presented to the participants [28]. To ensure the quality of the collected data, the data were screened and cleaned according to the IARC’s guidelines using an updated version of GD^®^ (0.2015.09.28) [36]. Afterwards, the foods, recipes, and ingredients obtained by the GD^®^ software were linked using the matching tool FoodCASE (Premotec GmbH, Winterthur, Switzerland) to the most suitable item found in the Swiss Food Composition Database [37].

Each menuCH participant was categorized into one of six alcohol consumption groups considering the participant’s pure alcohol intake from alcoholic beverages in the 24HDR as well as information on general alcohol avoidance from the self-administered questionnaire. Participants, who did not consume alcoholic beverages in the 24HDR and reported alcohol avoidance were categorized as ‘abstainer’, whereas participants who did not report alcohol consumption in the 24 HDR but did not report alcohol avoidance were categorized as ‘safe_no’. Participants, who did consume alcoholic beverages in the 24HDR were categorized into four groups. On the one hand, the categorization was based on whether the participants consumed more pure alcohol during or outside mealtime (‘during’ and ‘outside’ drinkers, respectively). Since the 24HDR did not directly assess the context of drinking, the definition by Sieri et al. [38] was used: an alcoholic beverage was consumed outside mealtime if during that specific time of the day less than 10% of the total daily energy intake (excluding energy intake from alcoholic beverages) was consumed and the consumed amount of pure alcohol was at least 5 g. On the other hand, the categorization was based on whether the participant drank on average more than the maximum daily recommendation (women: 12 g pure alcohol; men: 24 g pure alcohol) proposed by the EKAL [19]. If the participants drank on average more than recommended, they were categorized as heavy drinker (‘heavy’) and otherwise categorized as safe drinker (‘safe’). Therefore, the resulting six alcohol consumption groups were: ‘abstainer’, ‘safe_no’, ‘safe_during', ‘safe_outside’, ‘heavy_during’, or ‘heavy_outside’. An overview of the six alcohol consumption groups is given in the Online Resource Table S1.

To investigate diet quality, the alternate healthy eating index (AHEI) was calculated for each participant for each of the two 24HDR interviews [32]. Then, the average AHEI was determined and used in the analyses of alcohol consumption data. The AHEI is based on eleven components, each having a score between 0 and 10 points [32]; these components are: vegetables, fruits, whole grains, sugar sweetened beverages, nuts, meat, trans fat, long chain omega-3 fatty acids, polyunsaturated fatty acids (PUFA), sodium, and alcohol. We excluded the component ‘alcohol’ since the effect of alcohol consumption should only be contained in the alcohol group variable. Therefore, in the present study, AHEI scores between 0 and 100 were possible, with higher scores indicating a healthier diet.

### Swiss population and mortality data

The Swiss population census data and the mortality data were provided by the FSO [39]. For both databases, only residents between 18 and 75 years old were included to ensure the same age range as in the menuCH study. All-cause mortality and cause-specific mortality determined by the final cause of death (encoded using the 10th revision of the international classification of diseases (ICD-10) [40]) were investigated. The following cause-specific mortalities were investigated: CVD (ICD-10: I00-I99), all-cancer (ICD-10: C00-C97, D32-D33, and D37-D48), colorectal cancer (ICD-10: C18-C21), liver cancer (ICD-10: C22), upper aero-digestive tract (UADT) cancer (all organs and tissues of the respiratory tract, upper part of the digestive tract, and the upper esophagus (ICD-10: C00-C15 and C32), but excluding the stomach), breast cancer (only in women; ICD-10: C50), diabetes (ICD-10: E10-E14.9).

In addition, based on evidence for alcohol-related carcinogenic effects on human organs [17, 18], the following eight cancer sites were combined into one group: colorectal, liver, UADT, breast, prostate (ICD-10: C61), pancreatic (ICD-10: C25), urinary tract (ICD-10: C67-C68), stomach (ICD-10: C16) [41, 42].

Mortality ratios standardized for sex, age, and year of death (SMR) were calculated at the district level by dividing the number of observed deaths by the number of expected deaths in the overall Swiss population, based on the Swiss population census data. The latter was determined using an indirect method based on the standardized Swiss population mortality rates. The district of each menuCH participant was determined using their postal code and data provided by the FSO dated on the 1st of January 2019 [43, 44].

### Statistical analysis

Since not all Swiss inhabitants had the same probability to be included in the menuCH study sample, the analyses of the participants’ data of the final sample were weighted based on sex, age, marital status, major living region, nationality, household size, weekday, and season of the 24HDR day [27].

Descriptive statistics (absolute numbers, median, interquartile range, and percentages) were used to characterize the study population. Additionally, descriptive maps were used to show the geographic distribution of chronic diseases district-level SMR. The association between alcohol consumption and mortality was investigated by modeling mortality rates, fitting generalized linear regression models. Negative binomial regression models were used to handle overdispersion (for all-cause, CVD, all-cancer, and breast cancer mortality) and Quasipoisson models to handle underdispersion (for colorectal cancer, liver cancer, UADT cancer, and diabetes mortality). For each of the menuCH participants, the total number of observed deaths in the participant’s sex, age, and district category was used as outcome variable. The explanatory variable was the alcohol consumption group and the participant’s sex, age (continuous variable), smoking status, physical activity level, BMI group, education level, and average AHEI were included as further covariates. The log of the total number of residents stratified by the participant’s sex, age, and district category was included as offset term.

Missing values for physical activity level (*n* = 524), education level (*n* = 3), and smoking category (*n* = 4) (Table 1) were imputed using multivariate imputation by chained equations (MICE) [45]. The results of the 30 imputed data sets were pooled using Rubin’s rule [46]. In general, the results obtained with the imputed data sets were similar to the results of the complete case analyses. Therefore, only the results based on the imputed data sets are presented and used for further analyses.

**Table 1:** Characteristics of menuCH participants (*n* = 2057) stratified by alcohol consumption group^a,b^

Variable	Overall	Abstainer	Safe_no	Safe_during	Safe_outside	Heavy_during	Heavy_outside
*n*	2057	192	678	503	115	452	117
Women, *n* (%. %^*^)	1124 (54.6%, 50.2%)	135 (70.3%, 65.6%)	415 (61.2%, 56%)	252 (50.1%, 47%)	37 (32.2%, 27%)	232 (51.3%, 46.5%)	53 (45.3%, 42.7%)
Age (IQR)	45 (33, 58)	45 (35, 56)	41 (29, 54)	48 (35, 61)	40 (30, 53)	50 (40, 62)	40 (27, 54)
Age group, *n* (%, %^*^)							
18-29 years old	400 (19.4%, 18.8%)	40 (20.8%, 17.9%)	183 (27%, 25.9%)	63 (12.5%, 13.4%)	26 (22.6%, 20%)	48 (10.6%, 12.3%)	40 (34.2%, 30.7%)
30-44 years old	533 (25.9%, 29.9%)	50 (26%, 30.8%)	206 (30.4%, 34.4%)	127 (25.2%, 28.9%)	36 (31.3%, 38.1%)	83 (18.4%, 22.8%)	31 (26.5%, 30.5%)
45-59 years old	625 (30.4%, 29.8%)	62 (32.3%, 32.6%)	168 (24.8%, 23.1%)	169 (33.6%, 31.5%)	36 (31.3%, 28.2%)	162 (35.8%, 36.8%)	28 (23.9%, 23.4%)
60-75 years old	499 (24.3%, 21.6%)	40 (20.8%, 18.6%)	121 (17.8%, 16.6%)	144 (28.6%, 26.3%)	17 (14.8%, 13.7%)	159 (35.2%, 28.1%)	18 (15.4%, 15.3%)
Language region, *n* (%, %^*^)^c^							
German	1341 (65.2%, 68.8%)	112 (58.3%, 66.4%)	466 (68.7%, 70.6%)	326 (64.8%, 69.7%)	95 (82.6%, 81%)	256 (56.6%, 62.1%)	86 (73.5%, 75.6%)
French	502 (24.4%, 25.7%)	53 (27.6%, 26.5%)	151 (22.3%, 25%)	115 (22.9%, 22.8%)	15 (13%, 16.3%)	144 (31.9%, 32.5%)	24 (20.5%, 20.2%)
Italian	214 (10.4%, 5.6%)	27 (14.1%, 7.1%)	61 (9%, 4.4%)	62 (12.3%, 7.5%)	5 (4.3%, 2.7%)	52 (11.5%, 5.4%)	7 (6%, 4.2%)
Education level, *n* (%, %^*^)							
Primary	89 (4.3%, 4.7%)	15 (7.8%, 7.6%)	32 (4.7%, 5.8%)	21 (4.2%, 3.9%)	3 (2.6%, 1.7%)	13 (2.9%, 3.5%)	5 (4.3%, 3.8%)
Secondary	968 (47.1%, 43.2%)	93 (48.4%, 46.1%)	332 (49.2%, 44.9%)	229 (45.5%, 41.5%)	49 (42.6%, 41.1%)	205 (45.4%, 41.2%)	60 (51.3%, 47.1%)
Tertiary	997 (48.5%, 52.1%)	84 (43.8%, 46.2%)	311 (46.1%, 49.2%)	253 (50.3%, 54.6%)	63 (54.8%, 57.2%)	234 (51.8%, 55.3%)	52 (44.4%, 49.1%)
NA	3 (0.1%, 0.3%)	0 (0%, 0%)	3 (0.4%, 0.9%)	0 (0%, 0%)	0 (0%, 0%)	0 (0%, 0%)	0 (0%, 0%)
BMI (IQR)	24.3 (21.8, 27.1)	24.4 (21.5, 27.8)	24.0 (21.6, 26.8)	24.4 (21.9, 26.6)	25.7 (22.8, 28.1)	24.7 (22.0, 27.9)	24.4 (22.2, 26.8)
BMI group, *n* (%, %^*^)							
Underweight	51 (2.5%, 2.3%)	5 (2.6%, 2.6%)	18 (2.7%, 2.6%)	10 (2%, 1.9%)	4 (3.5%, 2.4%)	12 (2.7%, 2.4%)	2 (1.7%, 1.6%)
Normal	1115 (54.2%, 54.4%)	108 (56.2%, 57.2%)	395 (58.3%, 58.3%)	267 (53.1%, 55%)	53 (46.1%, 40.1%)	227 (50.2%, 50.7%)	65 (55.6%, 55.6%)
Overweight	629 (30.6%, 30.7%)	42 (21.9%, 19.3%)	191 (28.2%, 27.6%)	176 (35%, 34%)	42 (36.5%, 45.3%)	145 (32.1%, 33.5%)	33 (28.2%, 28.4%)
Obese	262 (12.7%, 12.5%)	37 (19.3%, 20.8%)	74 (10.9%, 11.4%)	50 (9.9%, 9.2%)	16 (13.9%, 12.2%)	68 (15%, 13.5%)	17 (14.5%, 14.4%)
Physical activity, *n* (%, %^*^)							
Low	219 (14.3%, 14.9%)	21 (15.1%, 24.1%)	78 (15.5%, 15.5%)	54 (14.1%, 13.7%)	8 (8.8%, 14.4%)	46 (13.6%, 12.6%)	12 (15%, 12.1%)
Moderate	487 (31.8%, 31.7%)	40 (28.8%, 23.7%)	148 (29.4%, 29.3%)	127 (33.2%, 34.2%)	28 (30.8%, 30.8%)	120 (35.6%, 36.6%)	24 (30%, 27.2%)
High	827 (53.9%, 53.4%)	78 (56.1%, 52.2%)	278 (55.2%, 55.3%	201 (52.6%, 52.1%)	55 (60.4%, 54.8%)	171 (50.7%, 50.8%)	44 (55%, 60.7%)
NA	524 (25.5%, 24.8%)	53 (27.6%, 29.1%)	174 (25.7%, 25.4%)	121 (24.1%, 22.3%)	24 (20.9%, 21.3%)	115 (25.4%, 25.2%)	37 (31.6%, 26.1%)
Smoking, *n* (%, %^*^)							
Never	914 (44.5%, 42.1%)	121 (63%, 64.1%)	332 (49.3%, 46.9%)	226 (44.9%, 42.9%)	55 (47.8%, 48.2%)	153 (33.8%, 30.1%)	27 (23.1%, 18.9%)
Former	688 (33.5%, 35.5%)	53 (27.6%, 29.2%)	202 (30%, 31.9%)	190 (37.8%, 40%)	35 (30.4%, 27.8%)	172 (38.1%, 40.4%)	36 (30.8%, 33.4%)
Current	451 (22%, 22.4%)	18 (9.4%, 6.8%)	140 (20.8%, 21.1%)	87 (17.3%, 17.1%)	25 (21.7%, 24%)	127 (28.1%, 29.6%)	54 (46.2%, 47.7%)
NA	4 (0.2%, 0.3%)	0 (0%, 0%)	4 (0.6%, 1.0%)	0 (0%, 0%)	0 (0%, 0%)	0 (0%, 0%)	0 (0%, 0%)
Pure alcohol intake [g/day] (IQR)	5.7 (0.0, 20.6)	0 (0, 0)	0 (0, 0)	8.0 (5.0, 11.8)	9.8 (6.4, 15.0)	30.7 (21.4, 43.8)	33.0 (25.1, 49.6)
AHEI (IQR)^d^	39.9 (31.7, 48.7)	42.4 (33.1, 52.3)	40.4 (32.1, 48.8)	40.7 (32.4, 49.5)	36.4 (29.4, 43.3)	39.0 (31.2, 47.1)	38.6 (30.2, 43.9)

*BMI* body mass index, *AHEI* alternate healthy eating index, *NA* missing values

^a^Participants not consuming alcoholic beverages in the 24HDR were categorized as ‘abstainer’ if reporting alcohol avoidance, and as ‘safe-no’ if not. Participants consuming alcoholic beverages in the 24HDR were categorized based on whether the participants consumed more alcohol during or outside mealtime (‘during’ and ‘outside’, respectively) and on whether their consumption was below or above the maximum daily recommendations (‘safe’ and ‘heavy’, respectively) [19, 38]

^b^Categorical variables are expressed as absolute number (*n*), unweighted percentage (%), and weighted percentage (%*). The weighted percentages (%*) are weighted according to the menuCH weighting strategy [27] for sex, age, marital status, major living region in Switzerland, nationality, household size, weekday, and season of the recall day. Continuous variables are expressed as weighted median and weighted interquartile range. The weighted median and IQR are weighted according to the menuCH weighting strategy [27] for sex, age, marital status, major living region in Switzerland, nationality, household size, weekday, and season of the recall day

^c^German language region: canton *Aargau*, *Basel City*, *Basel Country*, *Berne*, *Lucerne*, *Zurich*, and *St. Gallen*. French language region: canton *Jura*, *Neuchâtel*, *Vaud*, and *Geneva*. Italian language region: canton *Ticino*

^d^The AHEI is calculated as average AHEI of the two 24HDR. The AHEI is based on ten components each contributing between 0 and 10 points: vegetables, fruits, whole grains, sugar sweetened beverages, nuts, meat, trans fat, long chain omega-3 fatty acids, polyunsaturated fatty acids (PUFA), and sodium. The healthier the participant’s diet, the higher the AHEI score

The districts were defined as neighboring districts based on a first order neighborhood structure with rook contiguity. Additionally, the neighbors’ data were weighted by taking the inverse of the total number of neighbors of the corresponding district. The residuals of the regression models were investigated for spatial autocorrelation at the district level using global and local Moran’s *I*. The global Moran’s *I* statistic is an indicator for the existence and degree of spatial autocorrelation [47]. The statistic can range from -1 indicating spatial dispersion up to + 1 indicating spatial cluster-building [47]. A one-sided *P* value based on the Z-score was calculated [48]. To check the robustness, a one-sided *P* value based on 1000 Monte Carlo (MC) simulations was calculated additionally. Local Moran’s *I* were checked for significance based on a permutation test (*n* = 1000). No correction for multiple testing was included, since the number of Monte Carlo simulations determined the lower limit of the *P* value [49]. Local Moran’s *I* values were visualized using local indicators of spatial autocorrelation (LISA) cluster maps.

If evidence for spatial autocorrelation was detected, an integrated nested Laplace approximation (INLA) model was fitted. The structured spatial component was a *Besag* model [50] and the unstructured spatial component was an *iid* model (random noise). The default LogGamma prior distribution (shape = 1; rate = 0.00005) was used for both components. The results for each imputed data set were pooled by calculating the average of the estimates.

The analyses were performed in GeoDa (version 1.14.0) and in the R programming language (version 4.1.0; [51]). In R, the packages popEpi (version 0.4.8) and Epi (version 2.44) were used to calculate the SMR, mice (version 3.13.0) to impute missing values, survey (version 4.1.1) and DescTools (version 0.99.42) to conduct weighted analyses, MASS (version 7.3.54) to fit generalized linear regression models, spdep (version 1.1.8) and rgeos (version 0.5.5) to conduct spatial analyses, ggplot2 (version 3.3.5), ggsn (version 0.5.0), and sf (version 1.0.2) for creating figures, and INLA (version 21.11.22) to set up INLA models. For all analyses, the statistical significance level was set to 0.05.

## Results

### Descriptive results

The menuCH participants’ baseline characteristics stratified by alcohol consumption group are shown in Table 1. The largest group were the occasional drinkers (safe_no) with 678 participants and the two smallest were the two outside mealtime groups with 117 and 115 participants, respectively. All six alcohol consumption groups were characterized by differences across the variables investigated in comparison to the overall study population, e.g., abstainers and occasional drinkers were more likely to be never smokers, whereas during mealtime drinkers were more likely to be current or former smokers and outside mealtime drinking was more common among younger and during mealtime drinking among older participants. Median pure alcohol consumption per day stratified by sex and context of drinking can be seen in Fig. S2 and Fig. S3 (Online Resource).

**Fig. 1:**
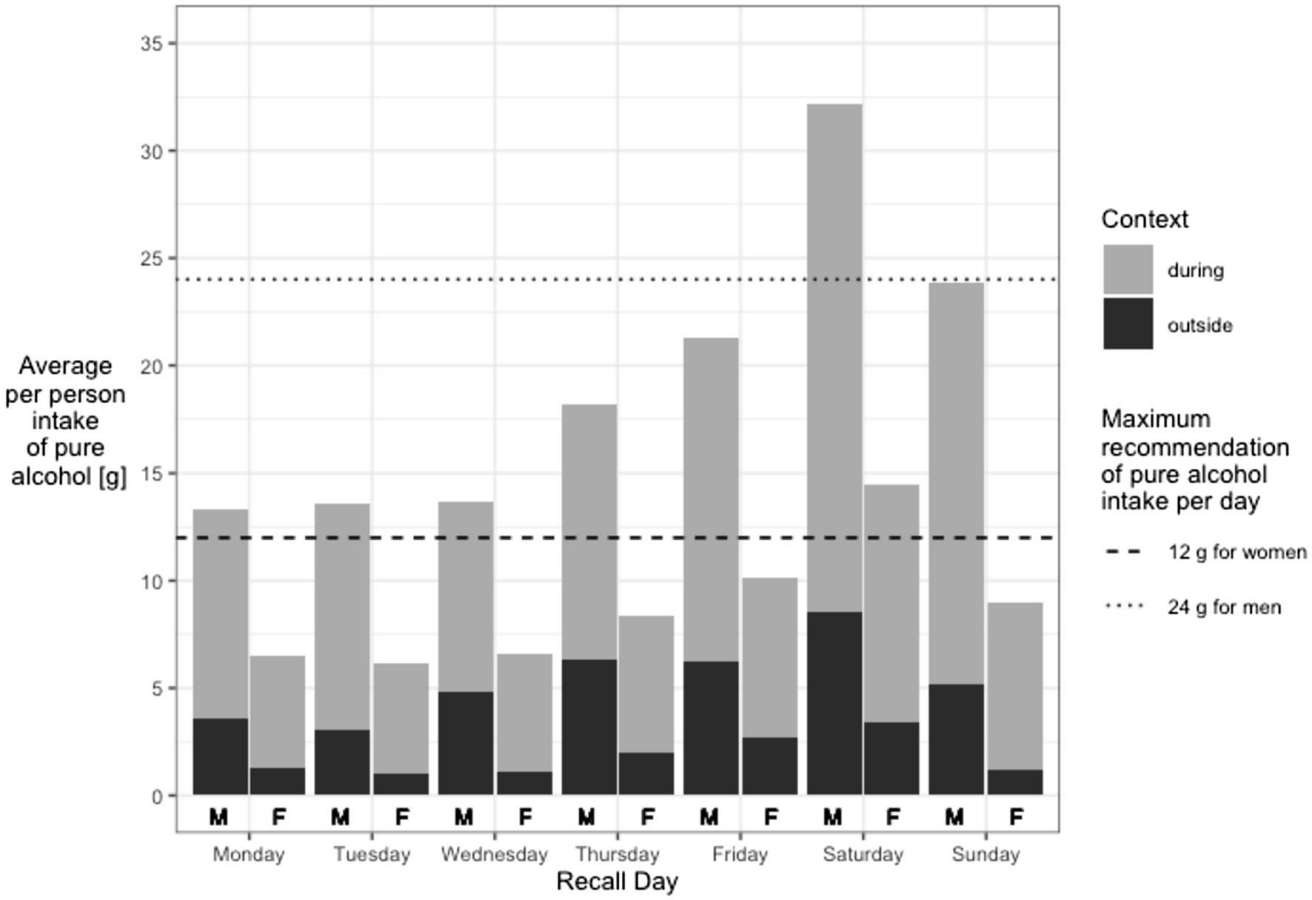
Average per person pure alcohol intake stratified by sex (M = male; F = female), recall day, and context of drinking (unweighted data, *n* = 2057). The threshold for pure alcohol intake per day proposed by the EKAL [19] is shown by a dashed line for women (12 g) and as a dotted line for men (24 g)

Figure 1 shows the average pure alcohol intake per person stratified by sex, weekday, and context of drinking. Regardless of weekday and context, men drank more than women. Both sexes consumed more pure alcohol during mealtime than outside. The amount of pure alcohol intake was lower at the beginning of the week, increased from Thursday onwards and reached its peak on Saturday. On Saturday, men consumed on average 23.7 g pure alcohol during and 8.5 g outside mealtime and women on average 11.1 g pure alcohol during and 3.4 g outside mealtime. On Saturday only, both sexes consumed on average more than the maximum daily recommendation given by the EKAL [19].

Between the years 2015 and 2018, the following number of deaths were documented in Switzerland: 84,959 all-cause deaths, 16,082 CVD deaths, 37,202 all-cancer deaths, 3327 colorectal cancer deaths, 1821 liver cancer deaths, 2360 UADT cancer deaths, 3064 breast cancer deaths among women, 1256 diabetes deaths, and 17,362 deaths attributable to the eight specific cancer sites group.

**Fig. 2:**
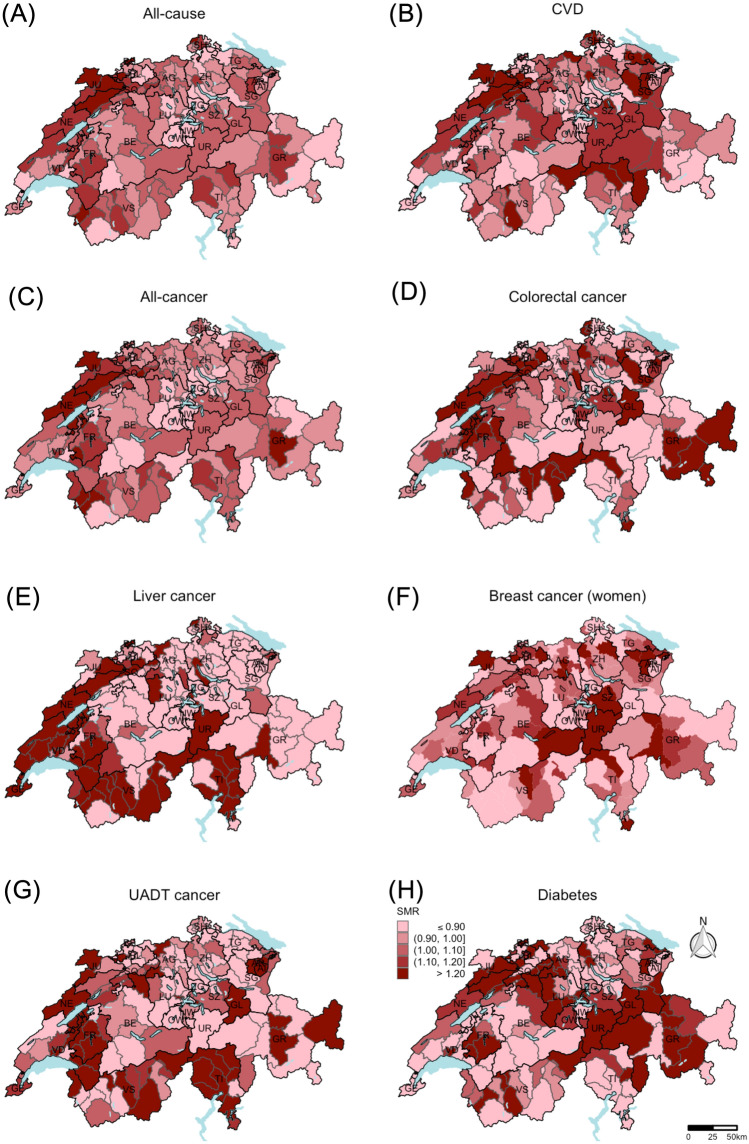
Standardized mortality ratios (SMR) at the district level (unweighted data, number of districts = 143). The SMR are standardized for sex, age, and year of death. The SMR were calculated using an indirect method with the whole Swiss population as reference population. Breast cancer SMR (F) were calculated only for women. For all other causes of death (**A**, **B**, **C**, **D**, **E**, **G**, **H**), the data of both sexes were included to calculate the SMR

The SMR at the district level are shown in Fig. 2. All-cause and all-cancer maps revealed a similar pattern with high SMR mainly in the western region and low SMR mainly in the central region. For CVD mortality high SMR were detected in the northwestern and eastern region. In contrast, many districts with low SMR were observed in the southwestern region. No clear pattern was detected for any of the specific cancer type mortalities, except for liver cancer: CH-French and CH-Italian districts tended to have higher SMR than CH-German districts. High diabetes SMR were more prevalent in central and northwestern region, whereas lower diabetes SMR were observed in the northeastern and southwestern region.

### Main results

Table 2 shows the results of the generalized linear regression models, with occasional drinkers used as reference group. In general, the consumption of alcoholic beverages tended to increase mortality rates, especially for all-cancer and UADT cancer mortality. Heavy, during mealtime drinkers had a higher risk of all-cancer (RR = 1.05, 95% CI 1.00, 1.10), breast cancer (RR = 1.10, 95% CI 1.00, 1.21), and UADT cancer (RR = 1.19, 95% CI 1.09, 1.31) mortality. Furthermore, heavy, outside mealtime drinkers had an increased UADT cancer (RR = 1.20, 95% CI 1.00, 1.43) and diabetes mortality (RR = 1.35, 95% CI 1.07, 1.71). Even for safe drinkers there was evidence for an increased risk of all-cancer (safe_during: RR = 1.07, 95% CI 1.02, 1.13), liver cancer (safe_outside: RR = 1.27, 95% CI 1.07, 1.51), and UADT cancer (safe_during: RR = 1.15, 95% CI 1.04, 1.26) mortality. Interestingly, the abstainers showed evidence for an increased all-cancer (RR = 1.07, 95% CI 1.00, 1.14) and UADT cancer (RR = 1.21, 95% CI 1.07, 1.37) mortality.

**Tab﻿le 2:** Association of alcohol consumption with sex-, age-, and district-specific mortality rate (*n* = 2057) (rate ratios and 95% confidence intervals)^a^

	All-cause^b,d,g^		CVD^b,d,g^		All-cancer^b,d,g^		Colorectal cancer^c,d,g^	
	RR	95% CI	RR	95% CI	RR	95% CI	RR	95% CI
Alcohol consumption group^f^								
Abstainer	1.01	0.96, 1.07	0.97	0.88, 1.07	1.07*	1.00, 1.14	0.97	0.88, 1.09
Safe_no (ref.)	1.00		1.00		1.00		1.00	
Safe_during	0.99	0.96, 1.03	0.99	0.93, 1.06	1.07^*^	1.02, 1.13	1.07	0.99, 1.16
Safe_outside	1.01	0.95, 1.08	1.01	0.90, 1.14	1.01	0.93, 1.11	1.15	0.99, 1.32
Heavy_during	1.00	0.96, 1.03	0.99	0.93, 1.06	1.05^*^	1.00, 1.10	1.05	0.97, 1.13
Heavy_outside	1.06	0.99, 1.13	1.07	0.95, 1.21	1.04	0.95, 1.13	1.08	0.93, 1.25

		Liver cancer^c,d,g^		Breast cancer^b,e,g^		UADT cancer^c,d,g^		Diabetes^c,d,g^	
		RR	95% CI	RR	95% CI	RR	95% CI	RR	95% CI
Alcohol consumption group^f^									
Abstainer		1.08	0.94, 1.24	1.10	0.97, 1.24	1.21^*^	1.07, 1.37	1.00	0.83, 1.20
Safe_no (ref.)		1.00		1.00		1.00		1.00	
Safe_during		0.96	0.87, 1.07	1.07	0.97, 1.18	1.15^*^	1.04, 1.26	0.93	0.81, 1.07
Safe_outside		1.27^*^	1.07, 1.51	1.20	0.95, 1.52	1.07	0.90, 1.28	1.04	0.81, 1.33
Heavy_during		1.09	0.99, 1.20	1.10^*^	1.00, 1.21	1.19^*^	1.09, 1.31	0.91	0.79, 1.04
Heavy_outside		0.96	0.79, 1.17	1.09	0.90, 1.32	1.20^*^	1.00, 1.43	1.35^*^	1.07, 1.71

*CVD* cardiovascular diseases, *UADT* upper aero-digestive tract, *RR* rate ratio, *CI* confidence interval

^a^The menuCH participants’ data were weighted according to the weighting strategy [27] for sex, age, marital status, major living region in Switzerland, nationality, household size, weekday, and season of the recall day

^b^A negative binomial regression model was fitted

^c^A Quasipoisson regression model was fitted

^d^The analysis included data of both sexes and were further adjusted for sex, age, smoking category, physical activity, BMI group, education level, and alternate health eating index

^e^The analysis included data of only women and was further adjusted for age, smoking category, physical activity, BMI group, education level, and alternate health eating index

^f^Participants not consuming alcoholic beverages in the 24HDR were categorized as ‘abstainer’ if reporting alcohol avoidance, and as ‘safe-no’ if not. Participants consuming alcoholic beverages in the 24HDR were categorized based on whether the participants consumed more alcohol during or outside mealtime (‘during’ and ‘outside’, respectively) and on whether their consumption was below or above the maximum daily recommendations (‘safe’ and ‘heavy’, respectively)[19, 38]

^g^Between the years 2015 and 2018, the following number of deaths were documented in Switzerland: 84,959 all-cause deaths, 16,082 CVD deaths, 37,202 all-cancer deaths, 3327 colorectal cancer deaths, 1821 liver cancer deaths, 3064 breast cancer deaths among women, 2360 UADT cancer deaths, and 1256 diabetes deaths

*Statistical significance (significance level $\alpha$= 0.05)

### Spatial analyses

**Fig. 3:**
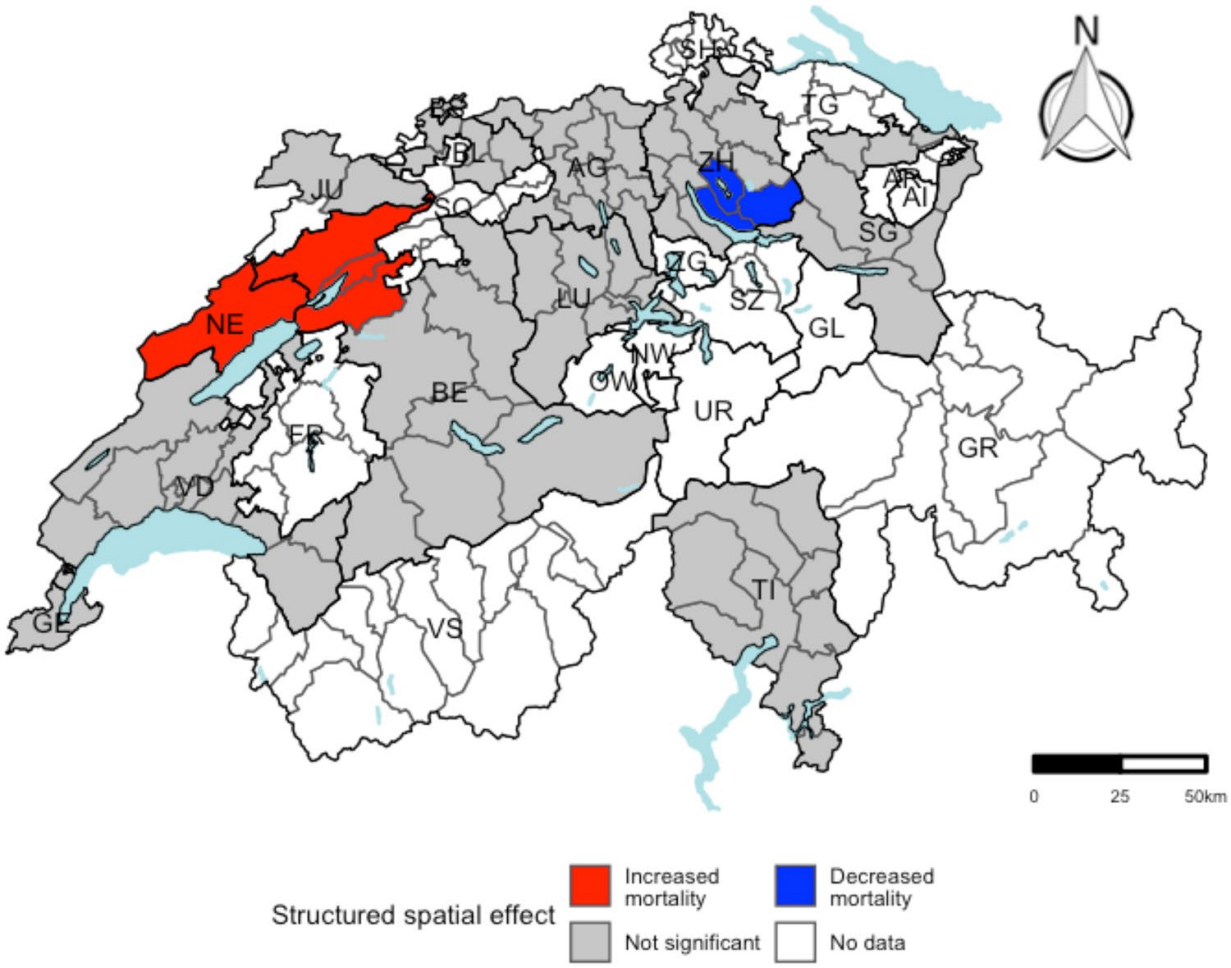
Geographic visualization at district level for the structured spatial component of the integrated nested Laplace approximation (INLA) model. Districts with a statistically significant structured spatial component are colored either red or blue, indicating significantly increased or significantly decreased mortality, respectively

The residuals of the generalized regression models were investigated for spatial autocorrelation at the district level using global Moran’s *I* statistic. The results of the spatial autocorrelation analysis are shown in Table 3. Only for all-cancer mortality, there was evidence for spatial autocorrelation (observed global Moran’s *I*: 0.144; expected global Moran’s *I*: − 0.014). The significant local Moran’s I values are visualized in a LISA cluster map (Online Resource Fig. S4). In total, 5 districts showed evidence for spatial clusters or spatial outliers (Fig. 3).

**Table 3:** Global Moran’s *I* statistic based on generalized linear regression model residuals at the district level (*n* = 75)

Cause of death	Observed Moran’s *I*	Expected Moran’s *I*	Variance Moran’s *I*	*P*_Z-score_^a^	*P*_MC_^a^
All-cause	– 0.013	– 0.014	0.007	0.5	0.44
CVD	0.101	– 0.014	0.007	0.086	0.11
All-cancer	0.144	– 0.014	0.008	0.035^*^	0.039^*^
Colorectal cancer	0.091	– 0.014	0.008	0.12	0.13
Liver cancer	– 0.056	– 0.014	0.007	0.3	0.28
Breast cancer	– 0.097	– 0.014	0.008	0.17	0.18
UADT cancer	0.016	– 0.014	0.001	0.22	0.18
Diabetes	– 0.032	– 0.014	0.005	0.4	0.44

*CVD* cardiovascular diseases, *UADT* upper aero-digestive tract, *MC* Monte Carlo

^a^One-sided *P* value with significance level $\alpha$= 0.05

*Statistical significance (significance level $\alpha$= 0.05)

Based on results of the INLA model (Table 4), there was evidence for an increased all-cancer mortality among abstainers (RR = 1.07, 95% CI 1.00, 1.14); safe, during mealtime drinkers (RR = 1.08, 95% CI 1.03, 1.13); and heavy, during mealtime drinkers (RR = 1.04, 95% CI 1.00, 1.09) compared to occasional drinkers.

**Table 4:** INLA model for association of alcohol consumption with sex-, age-, and district-specific all-cancer mortality: fixed effects (*n* = 2057) (rate ratios, standard deviations, 95% credible intervals)

	All-cancer^a^		
	RR	SD	95% CI
Alcohol consumption group^b^			
Abstainer	1.07^*^	0.03	1.00, 1.14
Safe_no (ref.)	1.00		
Safe_during	1.08^*^	0.02	1.03, 1.13
Safe_outside	1.02	0.04	0.94, 1.11
Heavy_during	1.04^*^	0.02	1.00, 1.09
Heavy_outside	1.05	0.04	0.97, 1.14

*RR* rate ratio, *SD* standard deviation, *CI* credible interval

^a^The negative binomial model part of the INLA model included data of both sexes and was further adjusted for the following fixed effects: sex, age, smoking category, physical activity, BMI group, education level, and alternate healthy eating index. The menuCH participants’ data were weighted according to the weighting strategy [27] for sex, age, marital status, major living region in Switzerland, nationality, household size, weekday, and season of the recall day

^b^Participants not consuming alcoholic beverages in the 24HDR were categorized as ‘abstainer’ if reporting alcohol avoidance, and as ‘safe-no’ if not. Participants consuming alcoholic beverages in the 24HDR were categorized based on whether the participants consumed more alcohol during or outside mealtime (‘during’ and ‘outside’, respectively) and on whether their consumption was below or above the maximum daily recommendations (‘safe’ and ‘heavy’, respectively) [19, 38]

*Statistical significance (significance level $\alpha$= 0.05)

Districts with a statistically significant structured spatial component are shown in Fig. 3. The districts *Uster* (RR = 0.91, 95% CI 0.82, 0.99), *Hinwil* (RR = 0.91, 95% CI 0.81, 1.00), and *Meilen* (RR = 0.89, 95% CI 0.78, 0.99) revealed evidence for a decreased all-cancer mortality rate, whereas the districts *canton Neuchâtel* (RR = 1.12, 95% CI 1.01, 1.27), *Jura Bernois* (RR = 1.13, 95% CI 1.01, 1.31), *Seeland* (RR = 1.14, 95% CI 1.01, 1.33), and *Biel* (RR = 1.18, 95% CI 1.01, 1.40) revealed evidence for an increased all-cancer mortality rate.

### Sensitivity analyses

The adjustments for diet quality via the AHEI did not meaningfully change the results of our study. The SMR for the cancer sites with evidence for a carcinogenic effect of alcoholic beverages [17, 18], which are not presented in Fig. 2, are shown in Online Resource Fig. S5. The results of the negative binomial regression model for mortality from the eight specific cancer sites were similar to those for all-cancer mortality (Online Resource Table S2). The global Moran’s *I* statistic revealed no evidence for spatial autocorrelation (observed Moran’s *I*: − 0.060, expected Moran’s *I*: − 0.014) and therefore, no INLA model was fitted.

## Discussion

In our study, descriptive differences in alcohol consumption were observed for anthropometric and lifestyle factors, revealing risk groups that should be targeted by alcohol-prevention strategies. Significantly higher mortality rates with increasing alcohol consumption were detected especially for all-cancer and UADT cancer, consistent with evidence-based carcinogenic effects of alcohol reported in previous studies [17, 18]. For the other investigated causes of death, the results pointed in the same direction, i.e., increase in mortality with increasing alcohol consumption, but were mostly not statistically significant. The INLA model for all-cancer mortality revealed Swiss districts with a significantly lower or higher all-cancer baseline mortality rate, indicating the existence of additional factors influencing all-cancer mortality.

Similarly as in a European study [38], alcohol consumption was higher for men than women, and higher during mealtime than outside. The amount of pure alcohol intake was lower at the beginning of the week, increased from Thursday onwards and reached its peak on Saturday. On Saturdays, both sexes consumed on average more than the maximum daily recommendation given by the EKAL [19]. This weekday pattern is in line with the current literature, which reports an increase in alcohol consumption toward the weekend and reaching a peak on Friday and Saturday [52, 53].

Observed differences in amounts and context of alcohol intake indicate that Switzerland has a similar general drinking pattern as other European countries. For example, a high amount of alcohol consumption was more prominent in men [19], in the CH-German and CH-French regions [19, 54], and in individuals with a high education level [19, 55], an increased, unhealthy BMI [56, 57], high physical activity level [58, 59], and in current smokers [60], compared with corresponding references. In our study, age differences were observed with respect to the context of drinking: younger participants were more prominent in the outside mealtime alcohol consumption groups, whereas older participants were more prominent in the during mealtime groups. Therefore, Switzerland could adopt an already existing and successfully implemented alcohol-prevention scheme of another country with a similar general drinking behavior.

The investigation of Swiss mortality data revealed a cause-of-death specific pattern at the district level. Regional variations such as the (diet) culture, socioeconomic factors, or urbanization of the districts might have influenced the SMR. Overall, the generalized linear regression models revealed a general trend of increased mortality rates across alcohol drinkers and abstainers compared to occasional drinkers. The resulting relationship was often a J-shaped curve that is commonly reported in the current literature for several causes of death [61]. Nevertheless, some researchers are questioning the J-shaped curve since the composition of the abstainer group is often heterogeneous, including former heavy drinkers or participants who avoid alcohol for reasons of poor health, and this leads to distorted results [62–64]. The increased rate among abstainers in our study might be due to the heterogeneous group composition, because the reason for alcohol avoidance was not assessed in the menuCH survey [64].

In our study, weak evidence was detected for an association of alcohol consumption with all-cause, CVD, and colorectal cancer mortality. In contrast, for all-cancer and UADT cancer mortality, strong evidence was observed for a higher mortality risk among abstainers and during mealtime drinkers in comparison to occasional drinkers. Overall, the highest rate ratios were observed in outside mealtime drinking groups for liver cancer, breast cancer, and diabetes mortality. Generally, the mortality rate ratios were all pointing in the same direction, indicating an increased mortality rate with increasing alcohol consumption, which is in line with the current literature on all-cause [63, 64] and noncommunicable disease mortality [17, 62, 65–71]. Therefore, our study suggests that there exists no safe alcohol drinking level. However, most rate ratios in our study were statistically not significant, presumably due to small sample sizes and small numbers of observed deaths.

Sieri et al. [38] postulated that alcoholic beverages might be more harmful when consumed outside mealtime. In our study, we found increased diabetes, UADT cancer, and liver cancer mortality rates for outside mealtime drinkers compared to occasional drinkers. In contrast, during mealtime drinkers had increased all-cancer, UADT cancer, and breast cancer (the latter only among heavy drinkers) mortality rates compared to occasional drinkers. Nevertheless, the outside and during mealtime alcohol consumption groups, when compared to occasional drinkers, revealed similar estimates overall. However, the latter does not imply that the context of alcohol consumption is not associated with mortality. Possible reasons for the lack of significance for alcohol consumption outside mealtime could be the low numbers of observed deaths, small sample sizes, and age differences among the alcohol consumption groups, leading to less observed deaths in alcohol consumption groups with mainly younger participants.

The Moran’s *I* statistic and the LISA map of all-cancer mortality indicated evidence for five districts to be spatial clusters and spatial outliers, respectively. The fixed effects estimates of the INLA model were similar to the estimates of the generalized linear regression model. The structured spatial component revealed evidence for an increased all-cancer mortality rate for four districts in cantons *Neuchâtel* and *Berne* and a decreased rate for three districts in canton *Zurich*. The detected geographic variation at the district level could have been caused by differences in (diet) cultures, urbanization of the districts, or socioeconomic factors. Further studies are needed to investigate the latter associations.

Our study had limitations that could have impacted the results. First, the drinking behavior of only two 24HDR was assumed to represent the general drinking behavior of each menuCH participant. In addition, since data sets were provided at different levels, our study assumed that the menuCH participants were correctly assigned to their district and were representative for their district’s alcohol consumption. Recall bias in the menuCH study could have led to over- or underestimation of alcohol consumption. Lastly, the menuCH study is not a longitudinal study but a cross-sectional study with possible reverse causation.

An important strength of our study was the survey weighting strategy, which enabled the 2057 menuCH participants to be representative for the target population. The postal code information enabled us to link the alcohol consumption data with mortality data. Moreover, the menuCH study provided information on all participants’ baseline characteristics, enabling us to adjust for known confounders. Lastly, the questionnaire enabled us to distinguish non-drinkers, who answered alcohol avoidance (abstainer), from those, who did not (safe_no).

In conclusion, significant associations of alcohol consumption with all-cancer and UADT cancer mortality were detected, indicating an increased mortality rate with increasing alcohol intake. For the other investigated causes of death, the results pointed in the same direction, but were statistically not significant. Significant spatial dependencies were observed for all-cancer mortality, revealing Swiss districts with evidence for a lower or higher all-cancer baseline mortality rate. Lastly, the present study highlighted important descriptive differences in alcohol consumption among sexes, age groups, education and physical activity levels, BMI, and smoking categories, revealing risk groups that should be the focus of future Swiss alcohol-prevention schemes. Therefore, the results of our study indicate the need for further studies to improve the next alcohol-prevention scheme and to lower the number of avoidable deaths in Switzerland.

## Supplementary Information

Below is the link to the electronic supplementary material.

Supplementary file1 (PDF 809 KB)

## Data Availability

The data and further documents of the menuCH study are available by request under https://menuch.iumsp.ch.

## Abbreviations

NCD: Non-communicable disease
CVD: Cardiovascular diseases
IARC: International agency for research on cancer
EKAL: Federal commission for alcohol issues
24HDR: 24-Hour dietary recall
FSO: Federal statistical office
BMI: Body mass index
WHO: World health organization
UADT: Upper aero-digestive tract
SMR: Standardized mortality ratio
MICE: Multivariate imputation by chained equations
LISA: Local indicators of spatial autocorrelation
INLA: Integrated nested Laplace approximation
MC: Monte Carlo
RR: Rate ratio
SD: Standard deviation
CI: Confidence interval and credible interval, respectively
NA: Missing values
AHEI: Alternate healthy eating index
