# Supplementary material for: Alcohol consumption: context and association with mortality in Switzerland
Source: Eur J Nutr. 2022 Dec 24;62(3):1331–44. doi: 10.1007/s00394-022-03073-w (PMC10030531; doi:10.1007/s00394-022-03073-w)
Supplement: Supplementary file 1 — Supplementary file1 (PDF 809 KB) [file 394_2022_3073_MOESM1_ESM.pdf]

# Supplemental Material

# Alcohol consumption:

# context and association with mortality in Switzerland


## Journal

European Journal of Nutrition



## Authors

Flurina Suter[1], Giulia Pestoni[1,2], Janice Sych[3], Sabine Rohrmann[1,*], Julia Braun[4]

## Affiliation

1 Division of Chronic Disease Epidemiology, Epidemiology, Biostatistics and Prevention Institute (EBPI), University of Zurich, Zurich, Switzerland

2 Nutrition Group, Health Department, Swiss Distance University of Applied Sciences, Zurich, Switzerland

3 Institute of Food and Beverage Innovation, ZHAW School of Life Sciences and Facility Management, Waedenswil, Switzerland

4 Divisions of Epidemiology and Biostatistics, Epidemiology, Biostatistics and Prevention Institute, University of Zurich, Zurich, Switzerland

*Correspondence: sabine.rohrmann@uzh.ch; Tel.: +41-446-345-256

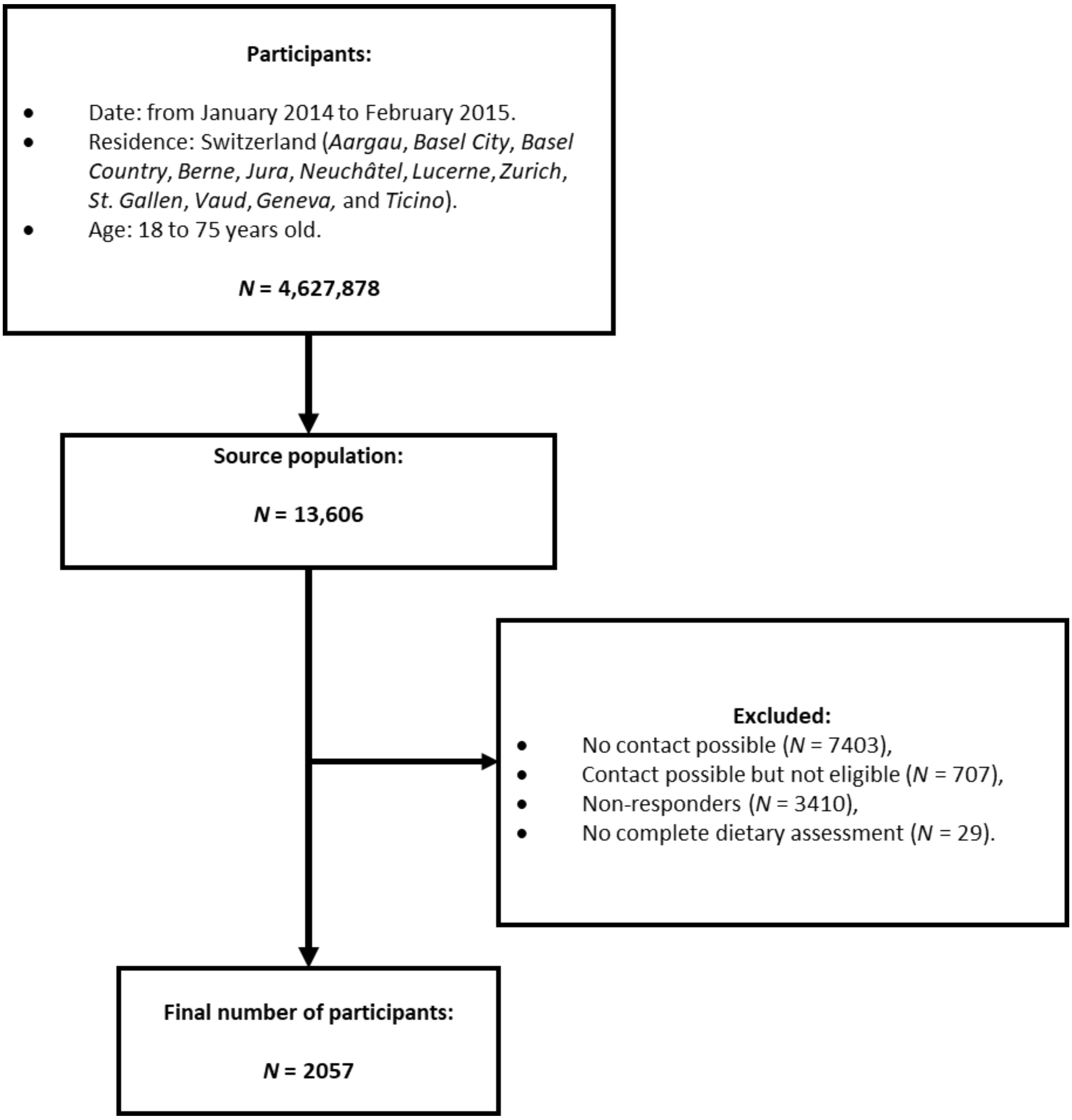


**Fig. S1** Flowchart of menuCH study population. Of the target sample of 4,627,878 Swiss residents a gross sample of 13,606 individuals were invited to participate in the menuCH study. 5496 individuals were successfully contacted, and 2086 individuals agreed to participate in the first 24-hour dietary recall (24HDR). Out of these 2086 participants, 2057 participants completed both 24HDR and were therefore used for the analyses. By applying the weighting strategy in the analyses, these 2057 participants represented the initial target sample of 4,627,878 Swiss residents [1]

Table S1 Alcohol consumption groups based on amount and context (during or outside mealtime) of consumption

| Group name | Description[a] |
|---|---|
| Abstainer | Participants reporting alcohol avoidance in questionnaire and not consuming alcohol in both 24HDR. |
| Safe_no | Participants reporting no alcohol avoidance in questionnaire and not consuming alcohol in both 24HDR. |
| Safe_during | Participants consuming not more than recommended by the EKAL and consuming ≥ 50% during mealtime. |
| Safe_outside | Participants consuming not more than recommended by the EKAL and consuming > 50% outside mealtime. |
| Heavy_during | Participants consuming more than recommended by the EKAL and consuming ≥ 50% during mealtime. |
| Heavy_outside | Participants consuming more than recommended by the EKAL and consuming > 50% outside mealtime. |

EKAL = Swiss federal commission for alcohol issues; 24HDR = 24-hour dietary recall

[a] Participants not consuming alcoholic beverages in the 24HDR were categorized as 'abstainer' if reporting alcohol avoidance, and as 'safe-no' if not. Participants consuming alcoholic beverages in the 24HDR were categorized based on whether the participants consumed more alcohol during or outside mealtime ('during' and 'outside', respectively) and on whether their consumption was below or above the maximum daily recommendations ('safe' and 'heavy', respectively) [2, 3]

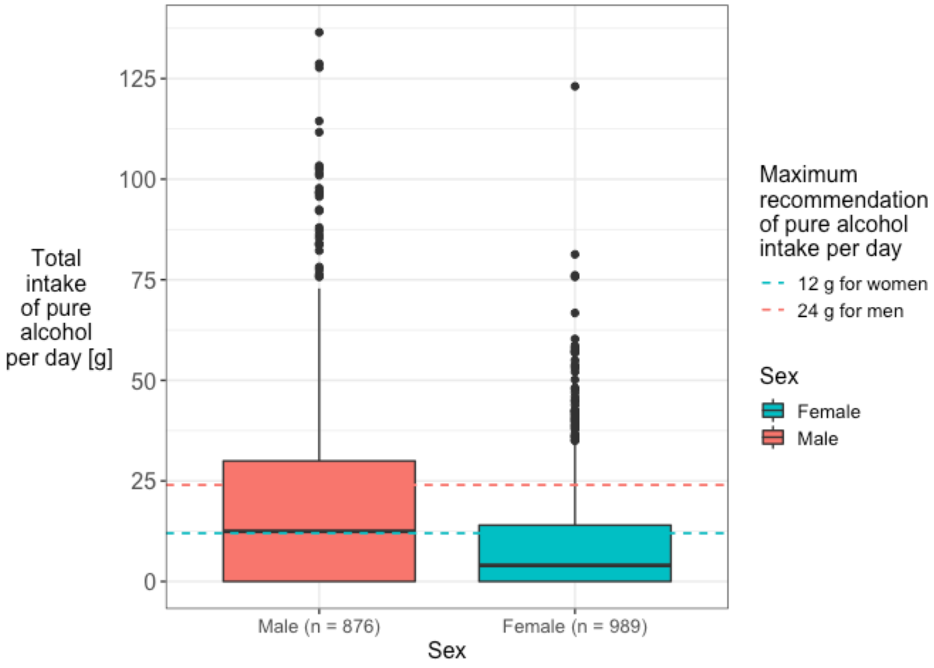


**Fig. S2** menuCH study participants' amount of pure alcohol consumed per day stratified by sex (unweighted data, *n* = 1865, excluding abstainers). The maximal daily recommendations proposed by the EKAL [2] are displayed as dashed lines (in red for men and in blue for women)

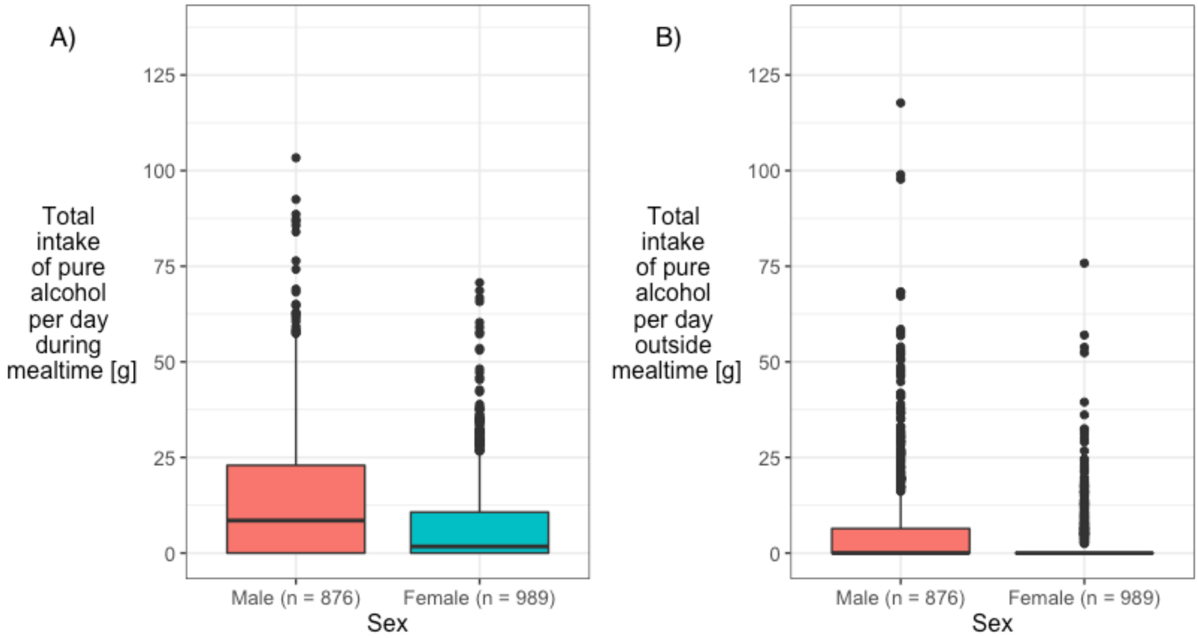


**Fig. S3** menuCH study participants' amount of pure alcohol consumed per day stratified by context of drinking (A: during mealtime; B: outside mealtime) and sex (unweighted data, *n* = 1865, excluding abstainers)

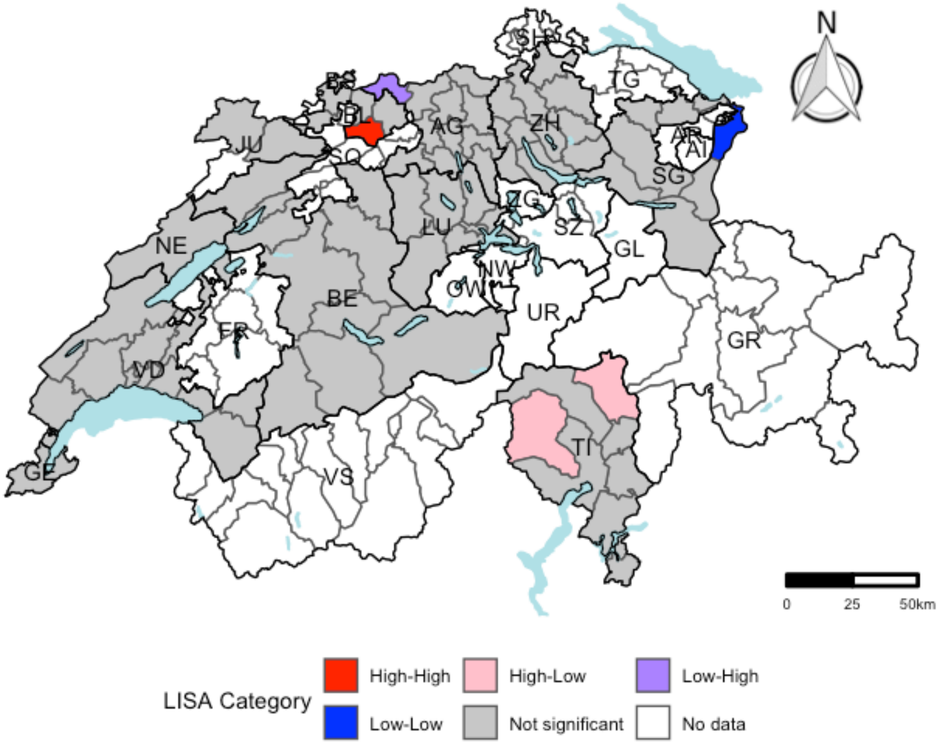


**Fig. S4** Visualization of local Moran's *I* for all-cancer mortality using a local indicators of spatial autocorrelation (LISA) cluster map at the district level (*n* = 75). 'High-High' represents districts which had a higher residual mean than the overall residual mean and a lagged value which was also higher than the overall mean lagged value. 'Low-Low' indicates districts for which both values were lower than the corresponding average value. 'High-Low' represents districts which had a higher residual mean than the overall mean and a lagged value which was lower than the overall mean lagged value and 'Low-High' represents districts for which the opposite was the case. Districts with a non-significant local Moran's *I* estimate are colored in gray. Districts which were not part of the menuCH study are colored in white. A significance level of 0.05 was applied

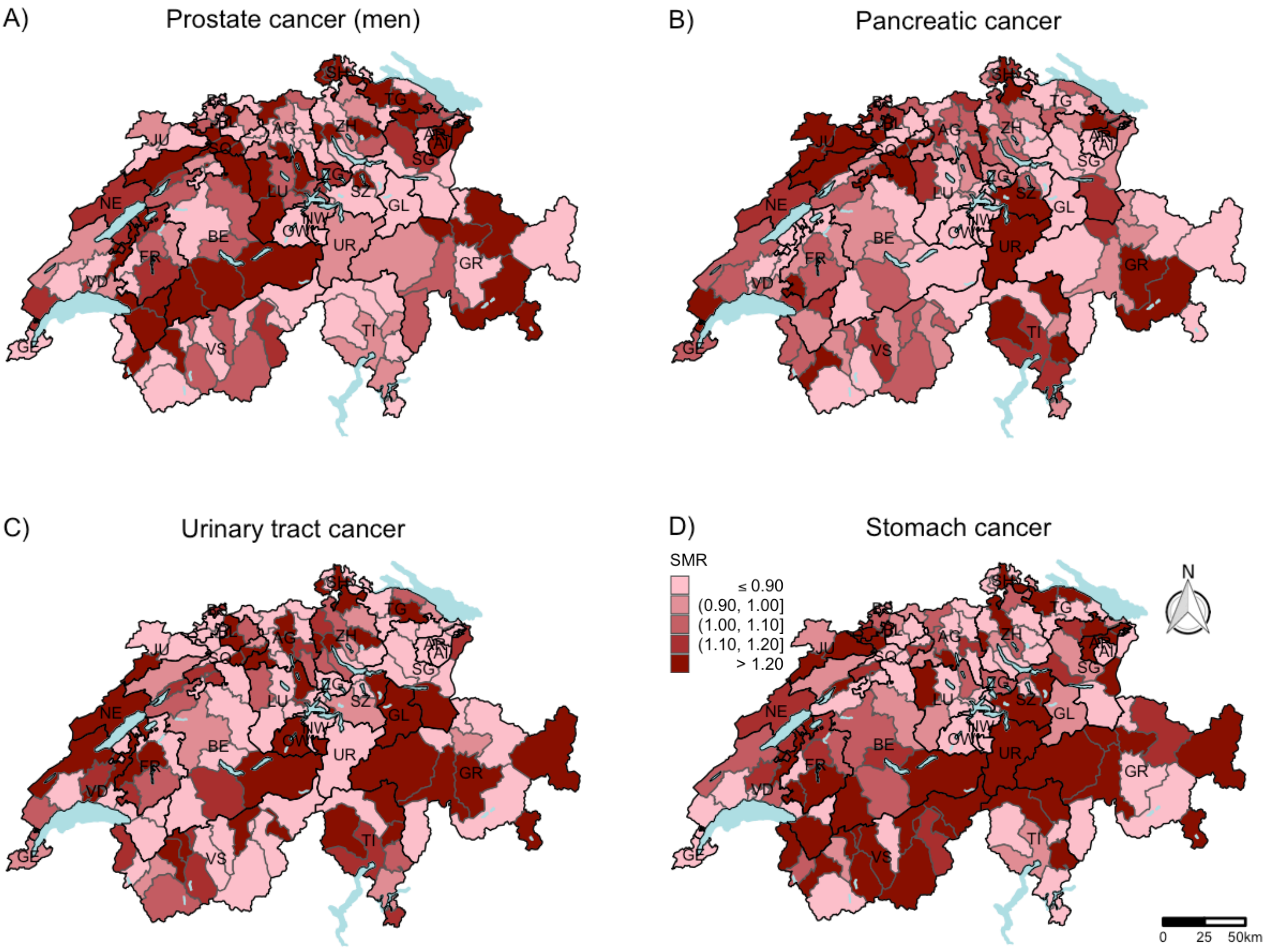


**Fig. S5** Standardized mortality ratios (SMR) at the district level (unweighted data, number of districts = 143). The SMR were standardized for sex, age, and year of death. The SMR were calculated using an indirect method with the Swiss population as reference population. Prostate cancer SMR (A) were calculated only for men. For all other causes of death (B, C, D), the data of both sexes were included to calculate the SMR

Table S2 Association of alcohol consumption with sex-, age-, and district-specific mortality rate (*n* = 2057) (rate ratios and 95% confidence intervals)

| | Eight specific cancer sites combined in one group[a] | |
|---|---|---|
| | RR | 95% CI |
| Alcohol consumption group[b,c] | | |
| Abstainer | 1.11* | 1.03, 1.21 |
| Safe_no (ref.) | 1.00 | |
| Safe_during | 1.08* | 1.01, 1.14 |
| Safe_outside | 1.06 | 0.95, 1.19 |
| Heavy_during | 1.08* | 1.02, 1.14 |
| Heavy_outside | 1.01 | 0.90, 1.13 |

RR = rate ratio; CI = confidence interval

[a] Eight specific cancer sites were combined in one group, as there is evidence for a carcinogenic effect of alcoholic beverages for these organs [4, 5]. The following cancer types were considered (encoded using the 10th revision of the international classification of diseases (ICD-10) [6]): colorectal cancer (ICD-10: C18-C21), liver cancer (ICD-10: C22), UADT cancer (all organs and tissues of the respiratory tract, upper part of the digestive tract, and the upper esophagus (ICD-10: C00-C15 and C32), but excluding the stomach), breast cancer (ICD-10: C50), prostate cancer (ICD-10: C61), pancreatic cancer (ICD-10: C25), urinary tract cancer (ICD-10: C67-C68), stomach cancer (ICD-10: C16) [7, 8]

[b] The menuCH participants' data were weighted according to the weighting strategy [9] for sex, age, marital status, major living region in Switzerland, nationality, household size, weekday, and season of the recall day. The negative binomial regression model included data of both sexes and was further adjusted for sex, age, smoking category, physical activity, BMI group, education level, and alternate health eating index

[c] Participants not consuming alcoholic beverages in the 24HDR were categorized as 'abstainer' if reporting alcohol avoidance, and as 'safe-no' if not. Participants consuming alcoholic beverages in the 24HDR were categorized based on whether the participants consumed more alcohol during or outside mealtime ('during' and 'outside', respectively) and on whether their consumption was below or above the maximum daily recommendations ('safe' and 'heavy', respectively) [2, 3]

* Statistical significance (significance level $\alpha$ = 0.05)

**References:**


1. Chatelan A, Beer-Borst S, Randriamiharisoa A, et al (2017) Major differences in diet across three linguistic regions of Switzerland: results from the first national nutrition survey menuCH. Nutrients 9:1–17. https://doi.org/10.3390/nu9111163
2. Federal Statistical Office (2019) BFS Aktuell—Alkoholkonsum 2017. Available at https://www.bfs.admin.ch/asset/de/10887924. Accessed 5 Apr 2022
3. Sieri S, Agudo A, Kesse E, et al (2002) Patterns of alcohol consumption in 10 European countries participating in the European Prospective Investigation into Cancer and Nutrition (EPIC) project. Public Health Nutr 5:1287–1296. https://doi.org/10.1079/phn2002405
4. International Agency for Research on Cancer (2021) List of classifications by cancer sites with sufficient or limited evidence in humans. IARC Monographs 1–130:1–14
5. Baan R, Straif K, Grosse Y, et al (2007) Policy Watch - Carcinogenicity of alcoholic beverages. Lancet Oncol 8:292–293. https://doi.org/doi: 10.1016/s1470-2045(07)70099-2
6. World Health Organization (2019) ICD-10 version: 2019 - International statistical classification of diseases and related health problems 10th revision. Available at https://icd.who.int/browse10/2019/en. Accessed 5 Apr 2022
7. Pestoni G, Karavasiloglou N, Braun J, et al (2022) Does diet map with mortality? Ecological association of dietary patterns with chronic disease mortality and its spatial dependence in Switzerland. Br J Nutr 127:1037–1049. https://doi.org/10.1017/S0007114521001525
8. Lohse T, Faeh D, Bopp M, Rohrmann S (2016) Adherence to the cancer prevention recommendations of the World Cancer Research Fund/American Institute for Cancer Research and mortality: a census-linked cohort. Am J Clin Nutr 104:678–685. https://doi.org/10.3945/ajcn.116.135020
9. Pasquier J, Chatelan A, Bochud M (2017) Weighting strategy. Available at https://menuch.unisante.ch/index.php/catalog/4/download/17. Accessed 5 Apr 2022
